# Beta-Mecaptoethanol Suppresses Inflammation and Induces Adipogenic Differentiation in 3T3-F442A Murine Preadipocytes

**DOI:** 10.1371/journal.pone.0040958

**Published:** 2012-07-23

**Authors:** Wen Guo, Yahui Li, Wentao Liang, Siu Wong, Caroline Apovian, James L. Kirkland, Barbara E. Corkey

**Affiliations:** 1 Department of Medicine, Boston University School of Medicine, Boston, Massachusetts, United States of America; 2 Robert and Arlene Kogod Center on Aging, Mayo Clinic, Rochester, Minnesota, United States of America; Pennington Biomedical Research Center, United States of America

## Abstract

Preadipocytes are present in adipose tissues throughout adult life that can proliferate and differentiate into mature adipocytes in response to environmental cues. Abnormal increase in adipocyte number or size leads to fat tissue expansion. However, it is now recognized that adipocyte hypertrophy is a greater risk factor for metabolic syndrome whereas fat tissue that continues to produce newer and smaller fat cells through preadipocyte differentiation is “metabolically healthy”. Because adipocyte hypertrophy is often associated with increased oxidant stress and low grade inflammation, both are linked to disturbed cellular redox, we tested how preadipocyte differentiation may be regulated by beta-mercaptoethanol (BME), a pharmacological redox regulator and radical scavenger, using murine 3T3-F442A preadipocytes as the cell model. Effects of BME on adipogenesis were measured by microphotography, real-time PCR, and Western analysis. Our data demonstrated that preadipocyte differentiation could be regulated by extracellular BME. At an optimal concentration, BME enhanced expression of adipogenic gene markers and lipid accumulation. This effect was associated with BME-mediated down-regulation of inflammatory cytokine expression during early differentiation. BME also attenuated TNFalpha-induced activation of NFkappaB in differentiating preadipocytes and partially restored TNFalpha-mediated suppression on adipogenesis. Using a non-adipogenic HEK293 cell line transfected with luciferase reporter genes, we demonstrated that BME reduced basal and TNFalpha-induced NFkappaB activity and increased basal and ciglitazone-induced PPARgamma activity; both may contribute to the pro-adipogenic effect of BME in differentiating F442A preadipocytes.

## Introduction

Impaired fat storage capacity in adipose tissue is implicated in the pathogenesis of obesity-related diseases. For instance, preadipocytes of type II diabetic subjects have been shown to have down-regulated expression of adipogenic genes, which could lead to reduced formation of adipocytes in fat depots, forcing excess fat storage in non-adipose tissue [1]. In addition, recent literature shows that drug-mediated inhibition of adipogenesis in mice on a high-fat diet results in significantly reduced weight gain and subcutaneous and gonadal fat mass but this effect was associated with marked adipocyte hypotrophy, enhanced macrophage infiltration, and apoptosis [2]. In contrast, compared to obese subjects with metabolic syndrome, metabolically healthy obese subjects have been shown to possess greater adipogenic activity and reduced inflammation [3]. Partly for this reason, it has been suggested that in the setting of obesity, an enhanced adipogenic capacity of fat tissue could be protective against other metabolic diseases [4].

To date, the causes that contribute to inhibit fat cell differentiation in metabolically unhealthy obesity are still not well understood. Low-grade chronic inflammation and associated cytokine production and oxidant stress have been shown to inhibit adipogenesis in general [5]–[7]. Based on the large body of literature showing increased oxidant stress in fat tissue of obese animals and humans [8], [9], one would expect that an increase of antioxidants in the cells may help to curb inflammation and improve fat tissue function, including restoration of active adipogenesis. Indeed, some studies have reported that selected antioxidants enhance adipogenesis [10], although others reported controversial findings [11]–[14].

Beta-mercaptoethanol is a strong sulfhydryl reagent that is widely used to improve growth and function of different cell types from different species [15]. As a thiol compound, BME can reduce extracellular cysteine to cystine, allowing the latter to re-enter the cells and serve as a precursor for GSH synthesis. GSH is the single most abundant anti-oxidant for detoxification and maintenance of appropriate thiolsulfide state for optimal cellular functions. Oral consumption of BME has been shown to prevent weight loss and even caused a moderate weight gain in aging rodent models [16]. These animals also lived longer and remained more active towards the end stage of life as compared with the controls [16]. Previous studies have shown that preadipocytes from aging animals generally lose their capacity for adipogenic differentiation [17]. This has been proposed as causal for aging-related ectopic fat store and insulin resistance [17]. It is tempting to speculate that BME might improve preadipocyte differentiation resulting in improved fat tissue and systemic health. For proof-of-principle, in this work we tested the effect of BME on adipogenic differentiation using 3T3-F442A murine preadipocytes.

Cell culture systems employing preadipocyte cell lines have been extensively used to study adipocyte differentiation. Murine 3T3-L1 and 3T3-F442A are two popular preadipocyte cell lines, both are subclones of 3T3 Swiss mouse embryo fibroblasts [18]. Unlike 3T3-L1 cells that require a stringent differentiation “cocktail” that contains high concentration insulin, dexamethasone, and 3-isobutyl-1-methylxanthine, differentiation of F442A cells can be induced by fetal bovine serum supplemented with a modest amount of insulin [19], [20]. Hence, F442A is considered to be at a more advanced stage in the mesenchymal to adipogenic linage. It has been shown that 3T3-F442A preadipocytes, but not 3T3-L1 cells, can differentiate into fat pads when injected subcutaneously into nude mice [21], [22]. Despite the difference in the induction protocols, both cell lines are well as primary preadipocytes undergo similar changes in molecular events during adipoctye differentiation, which involves the activation of peroxisome proliferator-activated receptor gamma (PPARgamma) and CCAAT/enhancer binding protein alpha (C/EBPalpha), which coordinate the subsequent expression of genes that changes the morphologic and metabolic phenotype of the cells [23]–[25]. In this work, we focused our studies on how BME might affect this differentiation cascade and explored a possible link between the pro-adipogenic and its anti-inflammatory roles of this thiol donor.

## Results

### BME Increases Lipid Droplet Accumulation in F442A Preadipocytes

**Figure 1:**
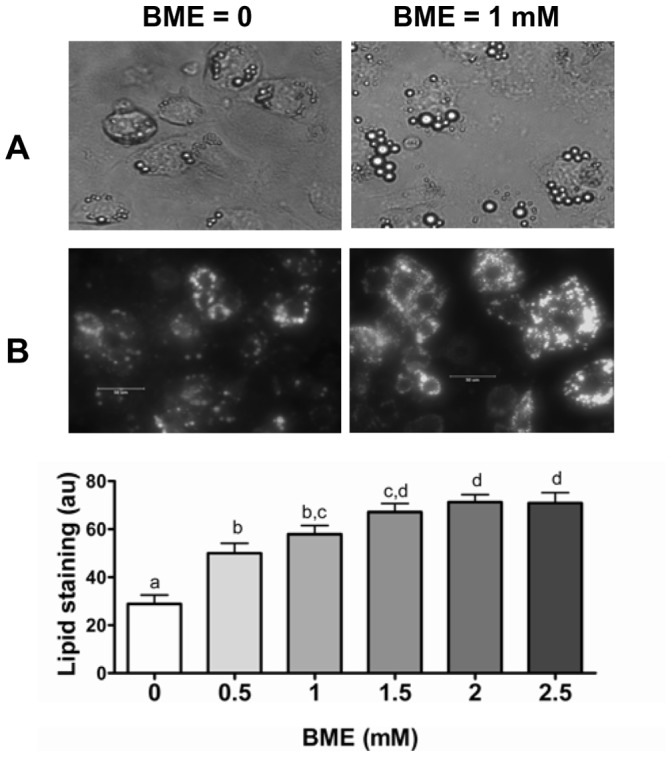
BME increases lipid accumulation in F442A cells. **A**: microphotograph of cells treated with control (left panel) and BME (1 mM, right panel) on day 6 after cells were induced to differentiated by insulin supplement. **B**: fluorescent microphotograph of cells after staining with BODIPY-C12 under otherwise the same conditions as those shown in A. bar = 5×10^−5^ m. **C**: quantitative fluorescent intensity of lipid staining on day 6. Results are mean +/− se from four independent cell cultures for each five different views were analyzed. Columns denoted with non-identical alphabets are statistically different (p<0.05, by Tukey’s test).

Upon confluence, F442A cells were treated with differentiation medium added with BME from 0–2.5 mM. Cell morphology was monitored daily by phase contrast microscopy. As shown in Figure 1A, cells treated with BME accumulated more lipids than the control cells. This was confirmed with quantitative analysis of lipid-associated fluorescent intensity after BODIPY staining [26], [27]. As shown in Figure 1B–C, BME increased the lipid incorporation of fluorescent BODIPY in a concentration-dependent manner and reached a plateau at 1–1.5 mM.

### BME Increases PPARgamma and C/EBPalpha Protein Expression

**Figure 2:**
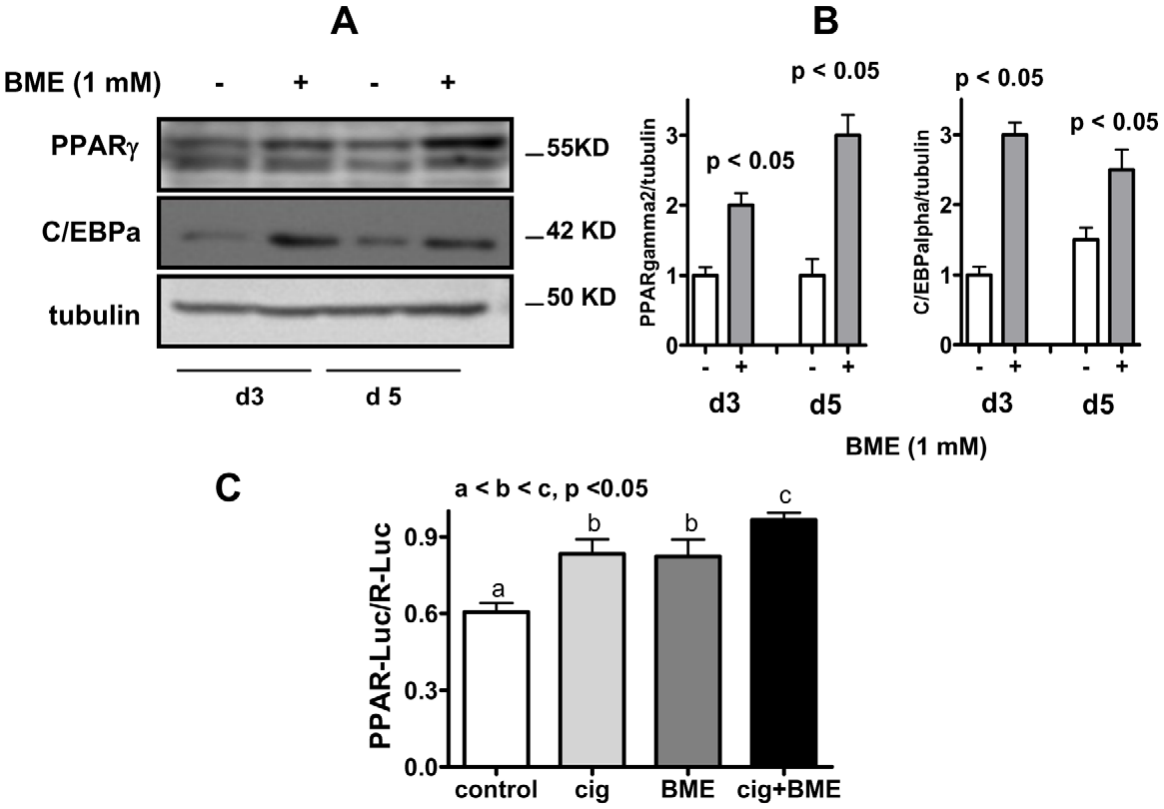
BME increases protein expression of PPARgamma and C/EBPalpha but does not directly regulate their transcription activities. **A**: representative blot of Western analysis for PPARgamma and C/EBPalpha in F442A cells differentiated without or with BME (1 mM). Cells were harvested on day 3 (d3) and day 5 (d5) after induced to differentiation by insulin and fetal bovine serum. **B**: quantification of PPARgamma and C/EBPalpha protein expression normalized to the loading control tubulin. Of note, although both PPARgamma1 (lower band) and PPARgamma2 (upper band) were detected, quantification was only done for PPARgamma2 because only this protein is adipocyte-specific. **C**: PPARgamma-driven luciferase activity in HEK293 cells treated with or without BME (1 mM) or ciglitazone (0.001 mM). Results are means +/− se (N = 3).

Adipocyte differentiation is regulated by the master transcription factor PPARgamma and its coordination with C/EBPalpha controls the expression of the majority of the metabolic genes involved in lipid synthesis and storage [28], [29]. As shown in Figure 2A–B, cells treated with BME expressed a significant increase in protein expression of both PPARgamma and C/EBPalpha at each time point measured. In addition, since PPARgamma contains multiple cysteine residues that may be subjected to regulation by thiol compounds or changes in cellular redox state [30]–[32], we also assessed whether BME might directly interacted with PPARgamma to regulate its activity, in addition to its effect on raising the protein mass of this transcription factor. To avoid the complex cross-regulations of multiple pathways within the context of a differentiating adipocyte, we studied the effect of BME on PPARgamma transactivation activity in HEK293 cells. Plasmids of recombinant mouse PPARgamma2 cDNA, PPARgamma cofactor RXRalpha, and PPARgamma reporter luciferase construct driven by a promoter containing three repeats of PPARgamma binding sites were co-transfected into HEK293 cells. After 24 h, cells were switched to high glucose DMEM containing insulin (200 ng/ml, same medium that was used for adipogenic differentiation) with and without BME (1 mM) or ciglitazone (0.001 mM). As shown in Figure 2C, ciglitazone and BME at their selected concentrations each moderately increased PPARgamma transcriptional activity. When added together, there was a moderate further increase in the activity. Treatment with BME did not increase the C/EBP-driven luciferase activity in HEK293 cells (data not shown). Together, these results suggest that BME directly modulate transcriptional activity of PPARgamma that may contribute to its pro-adipogenic effect.

### BME Increases Adipogenic Gene Expression

**Figure 3:**
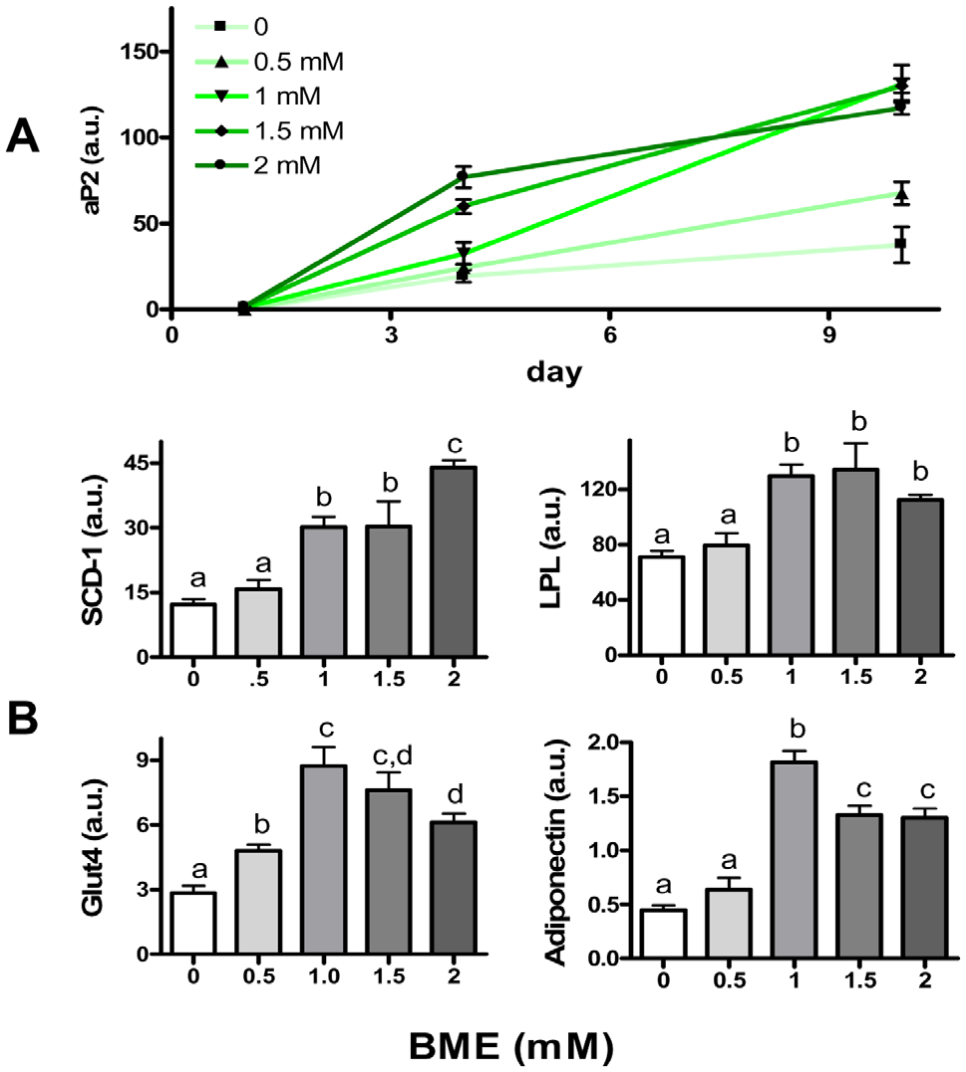
BME increases expression of adipogenic genes. **A**: The time course of aP2 mRNA expression in F442A cells after being induced to differentiate by insulin and fetal bovine serum and graded concentration of BME. **B**: Messenger RNA for SCD-1, LPL, Glut4, and adiponectin on day 10 of differentiation after being co-treated with graded concentration of BME. Results are means +/− SE (N = 3–6). Columns denoted with non-identical alphabets are statistically different (p<0.05, by Tukey’s test).

Coordinated induction of PPARgamma and C/EBPalpha is known to switch on the expression of a hierarchy of genes involved in adipogenic differentiation. Among these, adipocyte fatty acid binding protein (aP2) is one of the most prominent adipocyte-specific gene markers with its promoter containing multiple PPARgamma and C/EBPalpha binding sites [33]. Hence, the expression level of aP2 is often used as a generic marker for adipoctye differentiation [34]. As shown in Figure 3A, aP2 expression was minimal on day 1 after induction of differentiation. Subsequently, there was a time-dependent increase in aP2 expression in the control cells. At each time point tested, BME at each tested dosage was found to cause a significant increase in aP2 mRNA, suggesting that BME not only increased protein expression of PPARgamma and C/EBPalpha but also increased the transcription of their downstream adipogenic genes during preadipocytes differentiation. Expression of additional key elements in adipogenesis was also tested in cells harvested on day 10. As shown in Figure 3, BME increased expression of stearoyl-CoA desaturase-1 (SCD-1), lipoprotein lipase (LPL), and glucose transporter type 4 (Glut4). Moreover, BME was found to increase expression of adiponectin, an adipocyte-specific secreted hormone that has been implicated in positive metabolic regulation in liver and muscle [35]. It was noticed that, like aP2, BME increased expression of SCD-1 in a concentration-dependent manner. But for LPL, the effect reached a plateau after 1 mM whereas expression of Glut4, peaked at 1 mM of BME and was reduced with a further increase of BME, even though the expression level at 2 mM was still more than two fold higher than the control. Of note, although Glut4 has been characterized as one of the downstream gene targets of PPARgamma and C/EBPalpha [36], recent studies show that, in adipocytes, Glut4 promoter activity is also repressed by a transcription co-repressor histone deacetylase (HDAC). Since HDAC is generally activated by anti-oxidants, especially thiol compounds [37], the decrease of Glut4 expression at high concentrations of BME may be a result of thiol-induced HDAC activation. Further investigation is required to test this hypothesis.

### BME Modulates Expression of Inflammatory Cytokines in Differentiating Preadipocytes and Interacts with TNFalpha to Cross-regulate the Expression of Genes in the Adipogenic and Inflammatory Pathways

**Figure 4:**
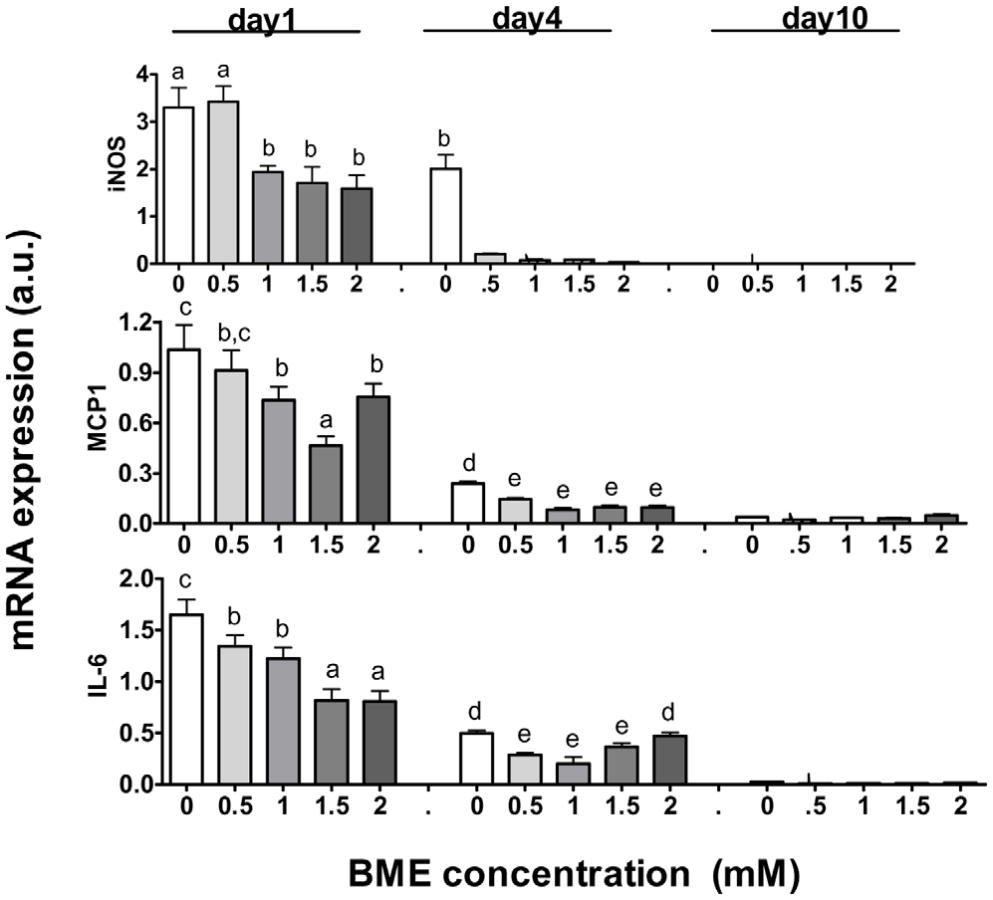
BME suppresses expression of inflammatory cytokines. Messenger RNA expression of selected cytokines on day 1, 4, and 10 after incubation with BME at graded concentration of BME. Results are means +/− SE (N = 3–6). Bars denoted with non-identical alphabets are statistically different (p<0.05, by Tukey’s test).

As a thiol compound, BME is well known for its strong anti-oxidant power, in part through its effect to increase synthesis of thiol-containing redox couples [38]. In light of the link between impaired preadipocyte differentiation and oxidant stress associated with fat tissue inflammation, we asked whether BME could regulate adipogenic differentiation through modulation of the inflammatory pathways. Using real-time PCR, we measured the effect of BME on the mRNA expression of selected inflammatory markers. As shown in Figure 4, the effect of BME was both time- and concentration-dependent. After 24 h incubation, BME reduced expression of Monocyte chemotactic protein-1 (MCP-1), Interleukin-6 (IL-6), and Inducible Nitric oxide synthases (iNOS), all well-established inflammatory cytokines and downstream targets of NFkappaB, the master transcription factor of inflammation. At this time point, BME was found not to increase expression of PPARgamma and C/EBPalpha, nor their downstream target genes (data not shown), implying that the anti-inflammatory effect of BME might occur temporally before its pro-adipogenic effect became detectable.

As shown in Figure 4, BME at 0.5–1 mM caused a sustained inhibition on cytokine expression throughout day 4 of the incubation period. For iNOS and MCP-1, this inhibition was maintained at higher BME concentration up to 2 mM. However, higher concentration of BME (>1 mM) was associated with a dose-dependent increase in IL-6 expression on day 4. At 2 mM, the expression of IL-6 was reversed back to the level of the control. The mechanism of this finding is not clear. A recent study shows that in vivo IL-6 is positively, while TNFalpha is negatively, related to thiol redox [39]. Hence, long-term exposure to high concentrations of BME may raise the thiol redox to increase IL-6 expression independent of its general anti-inflammation effect. Unfortunately, as with most other cell line models for preadipocytes, endogenous TNFalpha expression in F442A cells was very low and hence its response to exogenous BME treatment could not be accurately assessed. By day 10, the cells became fully differentiated and cytokine expression was decreased to negligible levels as compared to their original levels in the preadipocytes.

**Figure 5:**
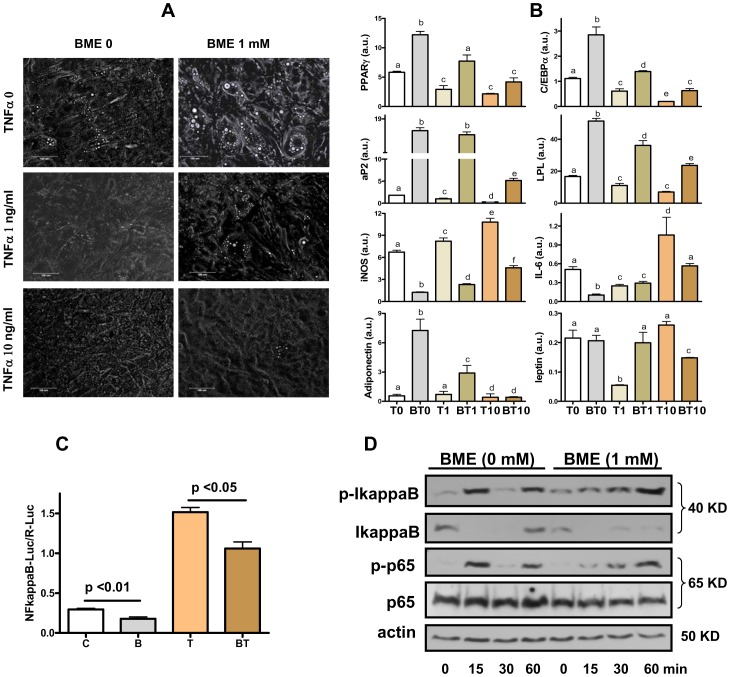
BME and TNFalpha mutually inhibits each other for effects on inflammation and adipogenesis. **A**: Phase-contrast microphotograph of F442A cells induced to differentiate by insulin for 9 days with or without co-treatment of BME and/or TNFalpha and indicated concentrations. **B**: Messenger RNA expression of selected marker genes for adipogenesis (PPARgamma, C/EBPalpha, aP2, and LPL), inflammation (iNOS and IL-6), and adipokines (adiponectin and leptin) in cells treated with TNFalpha at 0, 1 ng/ml, and 10 ng/ml without (T0, T1, T10) or with BME added at 1 mM (BT0, BT1, BT10). **C.** NFkappaB activity measured in HEK293 cells transfected with firefly reporter luciferase vector and control Renilla luciferase vector. Cells were treated with BME (1 mM) for 12 h before TNFalpha (10 ng/ml) was added without medium change and harvested after another 12 h of incubation (B: BME, T: TNFalpha, BT: BME plus TNFalpha). **D.** Western analysis for NFkappaB activation in response to TNFalpha (10 ng/ml) in differentiating preadipocytes. Cells were treated with differentiation medium with or without BME for 24 h. TNFalpha was then added directly to the culture and cells were harvested at different time points to perform Western analysis for p65^ser536^ and IkappaB^ser32^ and their corresponding total protein levels, using actin as the loading index. For A–C, results are means +/− SE (N = 3–6). For B, bars denoted with non-identical alphabets are statistically different, e.g. a ≠ b, etc. (p<0.05, by Tukey’s test). For D, results are representative of three independent repeated experiments.

To test if BME-induced adipogenesis was causally related to its modulation on inflammation, we co-treated the cells with BME together with TNFalpha, a pleiotropic cytokine known to induce oxidant stress and inhibit adipocyte differentiation [40], [41]. As expected, TNFalpha alone was found to reduce intracellular lipid accumulation (Figure 5A, left panel) in association with a strong inhibition on expression of PPARgamma and C/EBPalpha as well as their downstream genes aP2 and LPL (Figure 5B). This inhibitory effect was already significant at TNFalpha concentration of 1 ng/ml but was largely increased when the cytokine concentration was increased to 10 ng/ml (Figure 5A&B). While a low dose of TNFalpha alone did not drastically increase the IL-6 and iNOS expression, it was sufficient to partially blunt the BME-induced suppression on these cytokines. Of note, TNFalpha did not change the basal expression of adiponectin appreciably but reversed the induction of this adipokine by BME. Conversely, BME was found not to increase expression of leptin, another major adipokine. Interestingly, a low concentration of TNFalpha (1 ng/ml) reduced expression of leptin that was reversed by co-treatment with BME, likely a secondary effect to the changes in adipogenic differentiation. However, a high concentration of TNFalpha (10 ng/ml), while inhibiting adipogenic differentiation more potently, caused a slight increase in leptin expression as compared to control. While unexpected, this finding is consistent with prior studies documenting that TNFalpha induces leptin expression in adipocytes [42], [43]. Interestingly, the induction of leptin expression by TNFalpha was paradoxically blunted by BME. These data together indicate that BME and TNFalpha antagonizes each other in regulation of adipogenic differentiation and inflammation; lending additional support for the hypothesis that BME regulates preadipoctye differentiation and adipocytokine expression in part through modulation of the inflammatory pathways.

### BME Inhibits TNFalpha-induced NFkappaB Activation

NFkappaB is the best characterized redox-regulated transcription factor and also the master regulator for all the inflammatory cytokines measured in the above experiments [44]. To test whether BME directly regulates NFkappaB, we transfected a firefly luciferase reporter of NFkappB-Luc in HEK293 cells, using Renilla luciferase as control. As shown in Figure 5C, BME (1 mM) reduced both basal and TNFalpha-induced NFkappaB transactivation activity. Finally, we treated differentiating F442A cells with TNFalpha and measured its effect on NFkappaB activation. As shown in Figure 5D, 15 min after exposure to TNFalpha (10 ng/ml), there was a strong increase of phohspho-IkaapaB in the control cells, which was synchronized with the loss of IkappaB protein and increase of phospho-p65, an activated form of NFkappaB subunit. The response to TNFalpha was much weaker in the BME-treated cells, showing a mild increase in phospho-IkappaB and a milder increase in phospho-p65, although BME did not seem to delay the loss of IkappaB protein (Figure 5D). At 30 min, both phospho-IkappaB and phospho-p65 was reduced to minimum in the control cells. However, both signals reappeared strongly again in 60 min. Such an oscillatory pattern of NFkappaB activation in response to TNFalpha has been well documented in other cell types and mathematically modeled [45], [46]. The oscillatory pattern was not detected in the BME-treated cells within the time course of this experiment. Instead, we found a slower but continuous rise in both phospho-IkB and phospho-p65 with time, suggesting that BME delays TNFalpha signaling for NFkappaB activation in differentiating preadipocytes and dampens the maximal signal strength as well.

## Discussion

The key finding from this work is that BME, a thiol donor and radical scavenger, induced the adipogenic program in murine F442A preadipocytes, evidenced by increased protein expression of transcription factors PPARgamma and C/EBPalpha as well as the mRNA expression of their downstream target genes and established markers for adipocyte differentiation, including aP2, SCD-1, LPL, and Glut4. In association with its pro-adipogenic effect, BME rapidly reduced expression of inflammatory cytokines known to be downstream of NFkappaB, including MCP-1, IL-6, and iNOS. This effect was found to occur early during induction of adipogenic gene expression. Furthermore, BME interacted with exogenously added TNFalpha, a strong inducer for NFkappaB activation, partially blunting the effect of each other on adipocyte differentiation. These findings are not entirely surprising as the master transcription factors for adipogenesis (PPARgamma) and for inflammation (NFkappaB) are both thio-regulated proteins and they are also mutually inhibiting [30]–[32], [44], [47]–[53]. Mathematical modeling has predicted that whether a preadipocyte will differentiate or not is dependent on a dynamic interplay between PPARgamma and NFkappaB [54]. As expected, our data demonstrated that BME suppressed NFkappaB and activated PPARgamma both within the cell context of differentiating preadipocytes and in a non-adipogenic cell type. This would explain the findings of BME-mediated reduction of cytokine expression and up-regulation of adipogenic genes in differentiating preadipocytes.

Although NFkappaB is well known for its sensitivity to redox changes, how it is regulated by BME remains not completely understood. It has been shown that an increase in intracellular GSH inhibits TNFalpha-induced IkappaBalpha phosphorylation [55], [56], which is in good agreement with our current findings. However, there is also evidence that GSH can regulate NFkappaB activity through IkB-independent pathways [43], [49], [57], [58]. In agreement with these prior studies, we also noticed that exposure to TNFalpha for 15 min induced a similarly rapid loss of IkappB protein in both control and BME-treated cells, but phospho-p65 was markedly increased only in the control cells, implying additional mechanism by which BME impairs TNFalpha-induced phosphorylation (activation) of p65.

In addition to regulation of NFkappaB activity, changes in redox state can directly modulate other transcription factors and functional proteins, alter endoplasmic reticulum (ER) homeostasis, and even chromatin remodeling [59]–[61], all may have an effect on adipogenic differentiation. Many studies have documented that preadipocyte differentiation is inhibited by oxidant stress caused by either cytokines or free radicals [62], [63], in a way similar to our findings with BME. However, others reported controversial findings [12], [14], [64]–[70]. Of note, while BME is known to promote the reduction of cysteine to cystine, which is an important mechanism for intracellular GSH elevation, intracellular GSH levels and the GSH/GSSG ratio may increase or *decrease* with the addition of extracellular BME [71], [72]. In this work, we have not measured intracellular GSH or GSH/GSSG ratio in part because of the technical difficulty to prevent BME contamination to the cell lysates. By co-addition of BME with buthionine sulfoximine (BSO, 0.2–2 mM), an inhibitor for GSH synthase [14], we found no suppression of the pro-adipogenic effect of either (data not shown). Indeed, we found that BSO alone enhanced adipocyte differentiation (data not shown) which is in agreement with others’ reports [14]. Therefore, with the current data, we cannot draw a conclusion as to whether and how the changes in GSH or GSH/GSSG ratio *per se* mediate the BME-induced adipocyte differentiation under our experimental conditions.

Another interesting observation from this work is that we found a dramatic increase of adiponectin expression in cells treated with BME, an effect that was largely blocked by co-treatment with TNFalpha. While this effect could be secondary to the changes in adipocyte differentiation, BME may also have specific effects on this adipokine. It has been shown that adiponectin oligomerization is redox-dependent [73]. Whether this has any regulatory effect on its gene expression is not known. Besides, expression of leptin, another adipokine whose expression typically increases with differentiation, was found to respond to BME and TNFalpha in a very different manner from that of adiponectin. Hence, the changes in expression of these two adipokines may not be simply reflective of an overall stage of adipogenic differentiation. To date, adiponectin is one of the very few adipokines identified as positive regulators for systemic redox regulation, metabolism, and anti-inflammation [74]. Studies in humans have shown that short-term supplementation with antioxidant vitamins increases systemic adiponectin levels in both lean and obese subjects [75]. Our findings of BME-induced increase in expression of this anti-inflammatory adipokine coupled with its effect on expression of inflammatory cytokines and adipogenic genes can have useful clinical implications and is worth of further investigation.

In summary, this work provided novel evidence that BME, a thiol compound that may alter cellular redox state and scavenge reactive oxygen species, induced adipogenic differentiation in murine F442A preadipocytes, coupled with reduced expression of inflammatory cytokines and increased expression of anti-inflammatory adipokines. This effect was blunted by TNFalpha and nearly completely blocked by high concentration of TNFalpha. We suggest that BME may promote adipogenesis through its reciprocal effects on the master transcription factors NFkappaB and PPARgamma. Since “metabolically dysfunctional” obesity and aging are two well-established physiological conditions known to increase fat tissue inflammation and reduce preadipocyte differentiation [76], [77], and in light of recent studies showing that supplementation of BME in drinking water improves metabolic health and extends longevity in mice [15], [16], [78], it will be interesting to test our in vitro findings in animal models with impaired fat tissue plasticity, such as those with metabolic syndrome or at late stage of aging.

## Materials and Methods

### Cell Culture

Murine F442A preadipocytes were routinely grown and maintained in Dulbecco’s modified Eagle’s medium (DMEM) containing 10% iron-supplemented calf serum and standard antibiotics. Upon confluence, calf serum was switched to fetal bovine serum (FBS) (referred as day 0) and supplemented with insulin (200 ng/mL) and different concentrations of BME: 0–2.5 mM. Thereafter, medium was changed every other day and harvested at different time points from 24 h to 10 days. For selected experiments, murine TNFα (www.sigma-Aldrich.com) was added at 1 ng/ml and 10 ng/ml with or without BME. HEK293 cells were grown in DMEM supplemented with standard antibiotics and 10% FBS.

### Lipid Droplet Staining

Cells were stained with BODIOY as previously described. Briefly, BODIPY stock (1 mg/ml, in DMSO) was mixed to the differentiation medium (final 0.005 mg/ml) and used for cell incubation for 4 h. Cells were then washed with cold PBS containing 0.1% bovine serum albumin (BSA) for 4 times. After washing, the majority of the fluorescence was found to be associated with lipid droplets. The intracellular fluorescent intensity was then read on the plate reader at 530/550 nm.

### Transfection and Luciferase Activity Assay

Plasmids of PPARgamma2 and RXRalpha were a gift from Dr. BM Spiegleman (Harvard University). Plasmid for NFkappaB-Luc was described in our previous work [79]. Plasmids for luciferase reporter for PPARgamma and C/EBPalpha were purchased from Panomics Inc (www.panomics.com). Control plasmid for Renilla luciferase and Dual-luciferase assay kit were purchased from Promega (www.promega.com). Because HEK293 cells do not express PPARgamma, mixture containing plasmids of PPARgamma2 and its hetero-dimerizing partner retinoid X receptor alpha (RXRalpha), PPARgamma-Luc, and Renilla-Luc were co-transfected into the cells using lipofectamine following manufacturer’s instruction. For measurement of C/EBPalpha and NFkappaB activity, cells were co-transfected with C/EBP-Luc/Renilla-Luc or NFkappaB-Luc/Renilla-Luc only, since the cells express a significant amount of endogenous C/EBPalpha and NFkappaB. One day after transfection, Cells were switched to differentiation medium (insulin 200 ng/ml) with and without BME (1 mM) or ciglitazeone (0.001 mM) and harvested after another 24 h. For NFkappaB-luc assay, cells were switched to the same insulin-containing medium for 12 h. TNFalpha (10 ng/ml) was added directly to the culture medium and cells were harvested after another 12 h incubation in the presence of THFalpha and/or BME. Dual-luciferase activity was measured following the manufacturer’s instruction. The results were normalized to Renilla luciferase activity.

### Western Blotting Analysis

Cells were washed by cold PBS buffer and prepared for immunoblot analysis in a lysis buffer (50 mM Tris-HCl, Ph7.5, 1 mM EDTA, 1 mM EGTA, 10% glycerol, 1% Triton-X, 50 mM NaF, 5 mM Na pyrophosphate, 1 mM Na3VO4, 0.1%SDS, 1 mM PMSF, 1 mM DTT). Protein concentration was qualified with Bradford method (Bio-Rad laboratories, Inc. Hercules,CA), and equal amount of protein (50 ug) was separated by SDS-PAGE. Proteins were transferred to a polyvinylidene difluoride membrane and used for Western analysis following the stand protocol. Primary antibodies for PPARgamma and C/EBPalpha and tubulin and actin were purchased from Santa Cruz (Santa Cruz, CA). Primary antibody for IkappaB and phospho-IkappaB^ser32^, p65 and phospho-p65^ser536^ were from Cell Signaling (Danvers, MA). Secondary antibodies were obtained from Santa Cruz or Cell Signaling. Proteins were visualized using chemiluminescence reagent (Pierce, Rockford, IL) and quantified by densitometry.

### Real-time PCR Analysis

Total RNA was extracted using RNeasy mini kit (Qiagen, Valencia, CA). For reverse transcription, 1 ug of the total RNA was converted to first-strand complementary DNA in 20 ul reactions using AffinityScript Q-PCR cDNA Synthesis kit (Agilent Technologies, La Jolla, CA), which was subsequently diluted five times. For PCR sample preparation, 5 ul of cDNA was mixed in 20 ul reaction volume with 10 uM primer and SYBR master enzyme mix (SABiosciences, Frederick, MD). The reaction was initiated at 94°C for 10 minutes, followed by 40 cycles through 94°C 15 seconds and 60°C 1 minute. All reactions were performed in duplicate. All CT values were in the range of 20–30 cycles. Amplification curves were analyzed using SDS 1.9.1 software (Applied Biosystem, Forster City, CA). All measurements were run in duplicates and results were normalized to the expression of endogenous house-keeping gene hypoxanthinephophoribosyltransferase (HPRT).

### Statistical Analysis

Data are presented as means+/− SE. Comparison of means between two groups was performed using Student’s t-test. Comparison among multiple conditions was conducted by one way ANOVA followed with between group comparisons by Tukey’s test.
